# Outer Membrane Structural Defects in Salmonella enterica Serovar Typhimurium Affect Neutrophil Chemokinesis but Not Chemotaxis

**DOI:** 10.1128/mSphere.01012-20

**Published:** 2021-02-24

**Authors:** Eric J. Leaman, Alvin Aung, Alexie J. Jacques, Bahareh Behkam

**Affiliations:** a Department of Mechanical Engineering, Virginia Tech, Blacksburg, Virginia, USA; b Virginia Western Community College, Roanoke, Virginia, USA; c School of Biomedical Engineering and Sciences, Virginia Tech, Blacksburg, Virginia, USA; University of Kentucky

**Keywords:** *Salmonella*, chemokinesis, chemotaxis, host-pathogen interactions, microfluidic assay, neutrophils

## Abstract

Neutrophils, the first line of defense against pathogens, are critical in the host response to acute and chronic infections. In Gram-negative pathogens, the bacterial outer membrane (OM) is a key mediator of pathogen detection; nonetheless, the effects of variations in its molecular structure on the neutrophil migratory response to bacteria remain largely unknown. Here, we developed a quantitative microfluidic assay that precludes physical contact between bacteria and neutrophils while maintaining chemical communication, thus allowing investigation of both transient and steady-state responses of neutrophils to a library of Salmonella enterica serovar Typhimurium OM-related mutants at single-cell resolution. Using single-cell quantitative metrics, we found that transient neutrophil chemokinesis is highly gradated based upon OM structure, while transient and steady-state chemotaxis responses differ little between mutants. Based on our finding of a lack of correlation between chemokinesis and chemotaxis, we define “stimulation score” as a metric that comprehensively describes the neutrophil response to pathogens. Complemented with a killing assay, our results provide insight into how OM modifications affect neutrophil recruitment and pathogen survival. Altogether, our platform enables the discovery of transient and steady-state migratory responses and provides a new path for quantitative interrogation of cell decision-making processes in a variety of host-pathogen interactions.

**IMPORTANCE** Our findings provide insights into the previously unexplored effects of *Salmonella* envelope defects on fundamental innate immune cell behavior, which advance the knowledge in pathogen-host cell biology and potentially inspire the rational design of attenuated strains for vaccines or immunotherapeutic strains for cancer therapy. Furthermore, the microfluidic assay platform and analytical tools reported herein enable high-throughput, sensitive, and quantitative screening of microbial strains' immunogenicity *in vitro*. This approach could be particularly beneficial for rapid *in vitro* screening of engineered microbial strains (e.g., vaccine candidates) as the quantitative ranking of the overall strength of the neutrophil response, reported by “stimulation score,” agrees with *in vivo* cytokine response trends reported in the literature.

## INTRODUCTION

Neutrophils are the most abundant type of immune cell in humans, constituting up to 70% of leukocytes in circulation (1). These innate immune cells possess the capacity to sense a broad array of foreign and endogenous molecular signals (1). In their most prominent role as the “first responders” to sites of infection, chemical signatures common to many species of microbes, called pathogen-associated molecular patterns (PAMPs), are detected by neutrophils’ pattern recognition receptors (PRRs), including Toll-like receptors (TLRs) and formyl peptide receptors (FPRs). TLR stimulation leads to neutrophil activation and the production of inflammatory and bactericidal molecules, which greatly prolong the neutrophil’s life span and increase its chemokinesis (i.e., random migration in response to chemical stimulation) (2). On the other hand, FPRs belong to the class of G protein-coupled receptors (GPCRs) that mediate localization to the site of infection through chemotaxis (i.e., directed migration up a chemical gradient) (1). Together, TLRs and FPRs allow neutrophils to mount an effective antimicrobial response directly to live pathogens.

**FIG 1:**
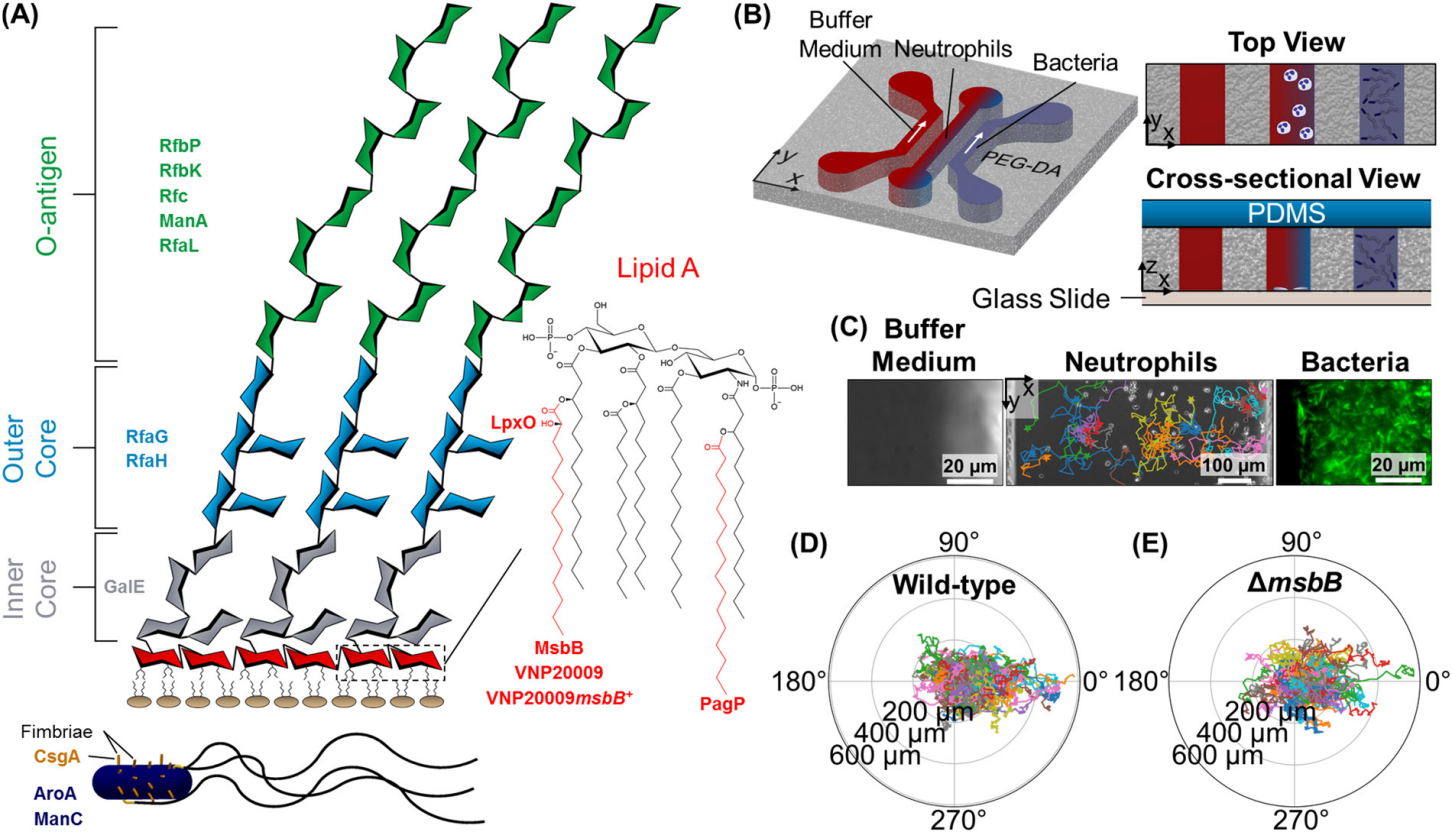
Analysis of the effects of outer membrane (OM) structure on neutrophil migration. (A) The molecular structure of the OM in *S.* Typhimurium and the corresponding proteins (or strain name in the cases of VNP20009 and VNP20009*msbB*^+^) encoded by the genes deleted in this study (3). The text color and location of protein names correspond to the specific component of LPS they modify. The proteins AroA and ManC (navy) have effects on multiple components. (B) The PEG-DA-based microfluidic device designed to physically separate bacteria and neutrophils while maintaining chemical communication between the two populations facilitates investigation of transient and steady-state migratory behavior in phagocytic cells. (C) Representative microcopy images showing a segment of the three channels of the device with trajectories of tracked neutrophils superimposed. (D and E) Polar plots showing trajectories of dHL-60 cells responding to wild-type and Δ*msbB* mutant bacteria, both located on the right-hand side of the cell channel (0°). The *x* axis was defined as being parallel to the gradient of diffusing effectors (as shown in panel B).

Of particular importance for the detection of Gram-negative bacteria, such as the model enteric pathogen Salmonella enterica serovar Typhimurium, is lipopolysaccharide (LPS), the primary component of the outer membrane (OM) and one of the best-characterized PAMPs. LPS is composed of the O-antigen polysaccharide on the outer surface, followed by a core oligosaccharide, connected to the hydrophobic anchor lipid A (Fig. 1A) (3). A high degree of neutrophil activation is caused by the binding of hexa-acylated, two-phosphate-group lipid A, such as that of wild-type *S.* Typhimurium. Modification to the OM structure results in less stimulation of the receptor (4). Attenuated strains have been produced by deleting LPS-related genes, resulting in less immunogenic molecular structures (5). It has also been reported that LPS-mediated TLR4 stimulation is not binary but highly gradated based on the structure of the LPS molecules (4). However, the relationship between the gradated stimulation of TLR4 and changes in the neutrophil activation and chemokinesis is unknown.

Costimulation of TLRs and chemotaxis receptors inadvertently occurs in the presence of live pathogens, and this is known to have complex effects on neutrophil migratory behavior. For instance, neutrophils lose their ability to effectively perform chemotaxis during sepsis, a host’s overwhelming inflammatory response to infection, presumably due to prolonged TLR stimulation (6). In contrast, TLR activation can either augment or weaken the chemotaxis response to various chemokines in less extreme scenarios (7, 8). It is, however, unclear how changes in chemokinesis due to TLR stimulation potentially affect chemotaxis.

Neutrophil chemotaxis *in vitro* is often studied using standardized assays such as the capillary assay (9), Boyden chamber (10), Zigmond chamber (11), or under-agarose assay (12). These assays have been crucial to advancing our understanding of the neutrophil’s migratory behavior; however, they produce only transient effector gradients and lack single-cell resolution. A plethora of studies investigating neutrophil chemotaxis and migratory behavior in microfluidic devices have been presented to address these issues (for a recent review, see reference 13). However, most of these studies have utilized purified effectors, e.g., *N*-formyl-Met-Leu-Phe (fMLP) or LPS, the results of which may not completely recapitulate the response to whole microbes. Few microfluidic platforms have combined neutrophils with live pathogens (14–17), wherein spatiotemporal variability in the interactions and the physical contact between the two populations present limitations. In particular, bacterium-neutrophil physical contact limits the total duration of experiments, preventing observation of the steady-state conditions that may be reached *in vivo* in settings such as chronic infections or tumors, where bacteria can be physically unreachable by neutrophils (18). Furthermore, neutrophil migration in response to attenuated pathogens or altered LPS structure is rarely explored. Thus, it is unknown how modulating the level of TLR stimulation could affect neutrophil chemokinesis and chemotaxis.

In the present study, we systematically addressed the question of how the neutrophils’ migratory responses (i.e., chemokinesis and chemotaxis) to live pathogens are affected by the OM structure of *S.* Typhimurium. We designed a microfluidic assay, which presents linear spatial gradients of effectors diffusing from live pathogens to neutrophils while maintaining physical separation, uniquely enabling a long-term O(hours) study of neutrophil behavior at single-cell resolution (Fig. 1B). We then screened the highly virulent *S.* Typhimurium 14028s and a library of 16 mutant strains harboring mutations in genes associated with OM structure and comprehensively characterized both the transient and steady-state neutrophil migratory responses. Not only does *S.* Typhimurium serve as a model Gram-negative pathogen, but its attenuated strains are among the leading candidates for bacteria-based cancer therapy (19), making a detailed understanding of their recruitment of immune cells doubly valuable. Using a host of quantitative metrics, we found significant differences in chemokinesis but little difference in chemotactic behavior of neutrophils in response to the various mutants, suggesting a lack of correlation between the two. Our results suggest that chemokinesis is more highly gradated than the chemotaxis response and that modification to any part of the OM structure could have profound or insignificant effects on the neutrophils’ response. To complement our biophysical study, we conducted neutrophil killing assays in human serum as a first indicator of how the mutations may affect survivability *in vivo*. Together, our results provide insight into how modifications to the wild-type OM affect innate immune cell recruitment and pathogen survival. These findings will enhance our understanding of host-pathogen interactions in infectious disease and will be useful for developing attenuated vaccine or cancer treatment strains. Our assay can also serve as a tool to interrogate the cell decision-making process in a variety of other host-pathogen interactions.

## RESULTS

We chose 16 mutant strains of *S.* Typhimurium 14028s with known or putative alterations in the outer surface of the bacteria (Fig. 1A; also, see Table S1 in the supplemental material). All but 4 of these strains contain single-gene deletions that are known to have a direct effect on the OM structure. These 4 strains include an Δ*aroA* mutant, which is presumably attenuated because of altered pathways that indirectly influence membrane structure (20), and the tumor-targeting strain VNP20009, which has a myriad of mutations stemming from a 108-kbp Suwaan deletion, purine auxotrophy, and altered lipid A structure due to the loss of the acyltransferase gene *msbB* (21, 22). Additionally, we evaluated VNP20009 with the *msbB* gene restored (VNP20009*msbB*^+^) for further insight into the role of *msbB* (23). Finally, we chose a Δ*csgA* strain as a first step to elucidating whether altered stimulation of a TLR other than TLR4 may also affect migration, as CsgA (a major constituent of fimbriae) is a TLR2 agonist (24).

10.1128/mSphere.01012-20.8 TABLE S1 Parental and mutant strains tested, affected protein(s), and functional results of the mutations. Download Table S1, PDF file, 0.08 MB. Copyright © 2021 Leaman et al. 2021 Leaman et al. https://creativecommons.org/licenses/by/4.0/ This content is distributed under the terms of the Creative Commons Attribution 4.0 International license.

As a surrogate for primary neutrophils, we used HL-60 human promyelocytic leukemia cells differentiated into a neutrophil-like phenotype (dHL-60s) in our experiments (see Materials and Methods for details) (25). We then compared our findings with results from primary human neutrophils responding to a subset of 9 of the 17 total strains, finding good agreement with the dHL-60 data.

### A microfluidic assay to quantitate neutrophil chemokinesis and chemotaxis behavior in response to live pathogens.

We developed a microfluidic platform based on previous work in our lab (26) to enable physical separation of neutrophils from live bacteria while allowing the diffusion of molecular signals (Fig. 1B). The device consisted of three parallel channels in the porous poly(ethylene glycol) diacrylate (PEG-DA) gel bonded to a glass slide and sealed on top by a layer of polydimethylsiloxane (PDMS). Given that the bottom (glass) and the top (PDMS) layers of the device are both liquid impermeable, the gradient generation was limited to the channel width, and the possibility of drift over time due to cross talk between the channels was minimized (27). Cells were introduced into the central channel, and the inlet and outlet were sealed to prevent flow. A bacterial suspension and a buffer medium were then flowed through the two outer channels for the duration of the experiment to develop a spatial gradient of the bacterium-derived effectors across the neutrophil observation channel and prevent the excessive buildup of neutrophil endogenous signals. We confirmed the diffusive transport of macromolecules through the PEG-DA using 10-kDa fluorescein isothiocyanate (FITC)-dextran (Fig. S1). Our experimental platform enabled three simultaneous experiments by fabricating three separate three-channel devices on a single chip. Time-lapse microscopy images of each neutrophil observation channel were recorded every 105 s over a period of 250 min. Cell migratory behavior was analyzed by tracking individual cells (Fig. 1C). Through a host of single-cell metrics, we quantitated and compared the chemokinesis and chemotaxis responses of cells stimulated with the wild-type and the mutant strains. In extreme cases, differences in response can be noted visually from cell trajectories. For instance, migration toward wild-type bacteria was more directed than migration in response to stimulation with the Δ*msbB* strain (Fig. 1D and E).

10.1128/mSphere.01012-20.1 FIG S1 Macromolecules effectively diffuse through 700-Da PEG-DA. (A) Bright-field micrograph showing the dimensions of the three-channel microfluidic device used to characterize 10-kDa FITC-dextran diffusion from the source channel to a sink (PBS) channel; (B) fluorescent intensity (relative to the source channel) as a function of lateral location in the observation channel; (C) fluorescence micrograph of the center channel at 10 h. Download FIG S1, EPS file, 0.7 MB. Copyright © 2021 Leaman et al. 2021 Leaman et al. https://creativecommons.org/licenses/by/4.0/ This content is distributed under the terms of the Creative Commons Attribution 4.0 International license.

### Variant LPS structures alter neutrophils’ chemokinesis.

**FIG 2:**
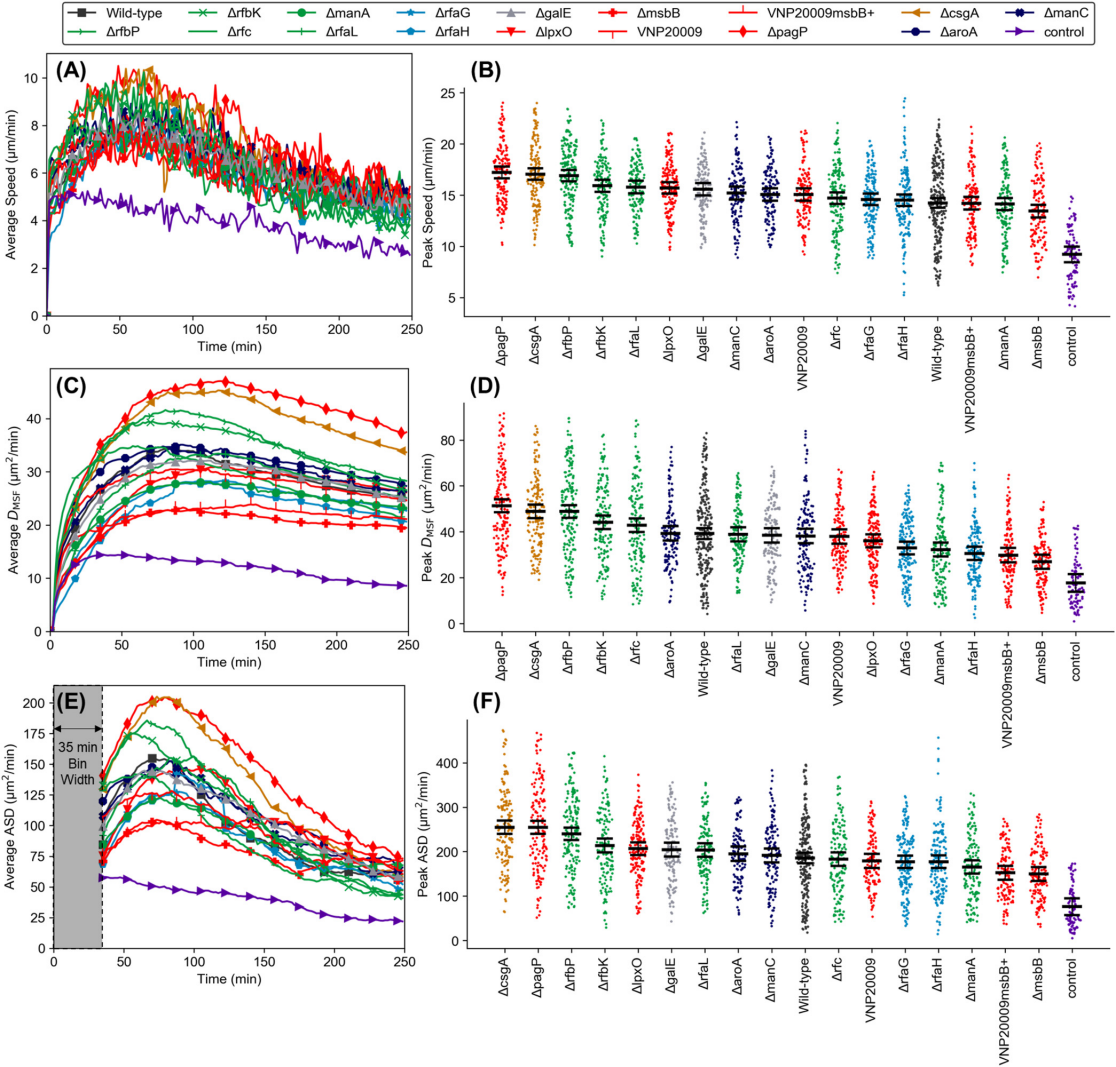
Dynamics of dHL-60 chemokinesis. (A) Averaged instantaneous cell speed versus time; (B) peak instantaneous speed for each tracked cell; (C) averaged effective cell diffusivity (*D*_MSF_) as a function of time; (D) peak *D*_MSF_ for each tracked cell; (E) average squared displacement (ASD) versus time; (F) peak ASD for each tracked cell. The results in panels B, D, and F are organized from left to right in order of highest to lowest average response. The black bars in panels B, D, and F indicate means and 95% confidence intervals (i.e., nonoverlapping bars between any two cases indicate a significant difference with a *P* value of <0.05). Data in panel E begin at 35 min based on the chosen bin width (shaded region). All results are from collections of at least 150 total cells tracked across three or more independent experiments (except the control case with 100 cells tracked across two experiments). Marker colors correspond to the specific component of OM that was modified, as shown in Fig. 1A.

We identified three metrics—instantaneous speed, diffusion coefficient, and average squared displacement—to comprehensively analyze changes in dHL-60 chemokinesis in response to bacterial stimulation for each of the 17 *Salmonella* strains. These three metrics were calculated for all the tracked dHL-60 cells responding to each bacterial strain. The average and peak values for these metrics are reported in Fig. 2.

The instantaneous speed was defined as follows:

(1) $$v=\frac{\sqrt{{\left(x_{i}-x_{i-1}\right)}^{2}+{\left(y_{i}-y_{i-1}\right)}^{2}}}{\Delta t}$$

where *x* and *y* denote spatial coordinates and Δ*t* is the time interval between two consecutive images. We calculated instantaneous speed first for all tracked cells at each point in time and averaged the results with respect to each bacterial strain (Fig. 2A) to evaluate the dynamic response in time. Speed-versus-time curves proved to be similar in shape but varied in magnitude. The noisiness of the data is expected, as rapid but small displacements are a hallmark of neutrophil activation (Movie S1). We next determined the maximum speed of each tracked cell (see Materials and Methods), finding significant differences between the average maximum speeds in response to different strains (Fig. 2B). Moreover, the maximum speed of cells was significantly higher in response to bacterial strains than the maximum speed in control experiments (no bacterial stimulation).

10.1128/mSphere.01012-20.10 MOVIE S1 Representative dHL-60 cell trajectories for strong and weak dynamic responses. This video shows the temporal evolution of the trajectories for dHL-60s tracked in one imaged region in experiments with highly stimulatory (Δ*rfbP*) and weakly stimulatory (Δ*msbB*) strains. Download Movie S1, MOV file, 13.9 MB. Copyright © 2021 Leaman et al. 2021 Leaman et al. https://creativecommons.org/licenses/by/4.0/ This content is distributed under the terms of the Creative Commons Attribution 4.0 International license.

As cells are known to exhibit random walk (or biased random walk in the presence of a chemo-effector gradient), we also sought to extract an effective diffusion coefficient (*D*_MSF_) of the migrating cells to describe and compare changes in the characteristics of the random walk in response to bacterial stimulation. We reasoned that this metric would effectively capture potential changes in dHL-60 chemokinesis due to gradated TLR4 stimulation. Since persistent motion toward the bacteria due to chemotaxis could affect this calculation, we adapted the mean displacement-mean square fluctuation (MD-MSF) analysis described in reference 28, which discounts net migration bias in the *x* and *y* directions:

(2) $$D_{\text{MSF}}\left(t_{N}\right)=\frac{1}{4\left(N-1\right)\Delta t}\overset{N-1}{\underset{i=1}{\sum }}\left[{\left(x_{i+1}-x_{i}-V_{x}\Delta t\right)}^{2}+{\left(y_{i+1}-y_{i}-V_{y}\Delta t\right)}^{2}\right]$$

where *t_N_* is the time at final time step *N*, Δ*t* is the time interval between successive images, and *x* and *y* describe the cell’s position, and

(3) $$V_{x}=\frac{x_{N}-x_{1}}{\left(N-1\right)\Delta t},\;V_{y}=\frac{y_{N}-y_{1}}{\left(N-1\right)\Delta t}$$

In this definition, *V_x_* and *V_y_* represent net cell speeds in the *x* and *y* directions, respectively. The *D*_MSF_ of dHL-60s exposed to bacteria increased rapidly at the start of an experiment until it reached a peak within about 100 min in most cases (Fig. 2C). In contrast, the *D*_MSF_ of dHL-60s in control experiments changed relatively little over the duration of an experiment. The *D*_MSF_ parameter had a higher signal-to-noise ratio than did speed, demonstrated by the smooth response curves, providing a clearer measure of the overall neutrophil response to particular mutants even at advanced points in time. Plotting the peak *D*_MSF_ values for all tracked cells demonstrated clear differences in the magnitude of responses to various mutants (Fig. 2D).

Finally, we defined a new metric that measures the average squared displacement (ASD) of a cell over discrete time intervals (i.e., a binned time duration). In contrast to the classical mean squared displacement (MSD) analysis, this analysis provided transient data while also reducing noise. The ASD is defined as follows:

(4) $$\text{ASD}\left(t_{N}\right)=\frac{1}{\left(N_{\text{bin}}-1\right)\Delta t}\overset{N}{\underset{i=N-N_{\text{bin}}}{\sum }}\left[{\left(x_{i}-x_{i-1}-V_{x,\text{bin}}\Delta t\right)}^{2}+{\left(y_{i}-y_{i-1}-V_{y,\text{bin}}\Delta t\right)}^{2}\right]$$

where *N*_bin_ is the number of time steps within the bin and *V_x_*_,bin_ and *V_y_*_,bin_ are the net cell speeds in the *x* and *y* directions, respectively, averaged over the binned period. Essentially, the ASD gives the rate of squared displacement of cells averaged throughout the binned period, which was chosen as 35 min (*N*_bin_ = 21). Plotting ASD versus time (Fig. 2E) demonstrated that the shape of the curves was qualitatively similar to that of speed curves, but the differences in responses between mutants were amplified. Measuring the peaks of each cell’s ASD demonstrated more gradated and statistically significant differences between many of the responses (Fig. 2F).

In all three analyses, the Δ*pagP*, Δ*rfbP*, and Δ*csgA* strains elicited the highest peak responses on average. These mutants had gene deletions in various OM components of lipid A (Δ*pagP*), O antigen (Δ*rfbP*), and fimbriae (Δ*csgA*), illustrating the complex compensatory mechanism in bacteria and unpredictable nature of neutrophil interactions with bacteria. While the average of each metric was significantly higher for each of these strains relative to a majority of other strains, the rank order and the significance of some differences changed between metrics. For instance, the speed and ASD responses to the Δ*lpxO* strain were similar in rank, being the sixth and fifth strongest responses, respectively, to all strains tested. For these metrics, the response to the Δ*lpxO* strain was not different from the response to the Δ*rfbK* strain. In contrast, the *D*_MSF_ was ranked twelfth for this strain, and the response to the Δ*rfbK* strain was indeed significantly stronger. Conversely, the responses to the Δ*rfc* strain demonstrate an opposite change in rank order. The average *D*_MSF_ values of dHL-60s responding to the Δ*rfbK* and Δ*rfc* strains were similar, but the average speeds and peak ASDs were significantly higher for the Δ*rfbK* strain. Such differences reveal the nature of the responses, being very rapid upon exposure to the Δ*rfc* strain but also quickly decaying, while being delayed but sharp and overall more sustained over time for the Δ*lpxO* strain (Fig. S2A to C).

10.1128/mSphere.01012-20.2 FIG S2 The nature of the average dynamic responses of dHL-60 cells. (A to C) Speed (A), effective cell diffusivity (*D*_MSF_) (B), and average squared displacement (ASD) (C) versus time in response to Δ*lpxO* and Δ*rfc* strains. (D to F) Speed (D) *D*_MSF_ (E), and ASD (F) versus time in response to Δ*rfbP* and Δ*rfaL* strains. Each curve is averaged across all tracked cells. These data exemplify how the dynamic nature of the responses can differ leading to large differences in the ranked order of results between the metrics. Note that speed data in panels A and D were smoothed using a moving average of every 11 data points to reduce noise for clarity. Download FIG S2, EPS file, 0.2 MB. Copyright © 2021 Leaman et al. 2021 Leaman et al. https://creativecommons.org/licenses/by/4.0/ This content is distributed under the terms of the Creative Commons Attribution 4.0 International license.

For other strains, rank order remained similar between metrics, although the significance levels changed between responses. For instance, the Δ*rfbP* strain did not invoke significantly higher peak speed than the Δ*rfaL* strain, but it did cause significantly higher peak *D*_MSF_ and ASD values. In this case, the evolution of all three metrics with respect to time was similar but higher in magnitude for the Δ*rfbP* strain (Fig. S2D to F). The Δ*rfaH* and Δ*manA* strains and, interestingly, both VNP20009*msbB*^+^ and the Δ*msbB* strain elicited the four weakest responses of all strains for all metrics. From this comprehensive analysis of dHL-60 dynamic behavior, we can glean that the Δ*rfaH*, Δ*manA*, VNP20009*msbB*^+^, and Δ*msbB* strains were indeed most attenuated with respect to dHL-60 dynamic behavior and that the Δ*rfaL* strain is less attenuated than these but more attenuated than the Δ*rfbP* strain. Lastly, Δ*rfc* and Δ*lpxO* strains each cannot necessarily be considered more attenuated than the Δ*rfbK* strain. In addition to experiments with bacteria, we performed positive-control experiments with various concentrations of the purified bacterial effectors fMet-Leu-Phe (fMLP) and complement component 5a (C5a), alone and in combination (Fig. S3A). We found that several bacterial strains stimulated significantly greater chemokinesis than these purified effectors. Furthermore, we observed larger differences in responses to the bacterial strains than to the order-of-magnitude changes in effector concentrations (Fig. S3B).

10.1128/mSphere.01012-20.3 FIG S3 dHL-60 chemokinetic and chemotaxis response to fMLP and C5a. The peak ASD in response to each of the purified effector concentration tested (A) and all results for relative comparisons (B), and the peak DP after the first 5 min of data collection in response to each purified effector concentration (C) and all results for relative comparisons (D). Error bars denote 95% confidence intervals (i.e., nonoverlapping bars between any two cases indicate a significant difference with a *P* value of <0.05). All results are from collections of at least 150 total cells tracked across three or more independent experiments (except the control and the 10 nM+C5a cases, with 100 cells tracked across two experiments each). Cells with a starting *x* of <210 μm were excluded from the DP analysis. Download FIG S3, EPS file, 0.6 MB. Copyright © 2021 Leaman et al. 2021 Leaman et al. https://creativecommons.org/licenses/by/4.0/ This content is distributed under the terms of the Creative Commons Attribution 4.0 International license.

Our results suggest that the expression of genes involved in OM biosynthesis and assembly significantly alters the migratory behavior of dHL-60s. The Δ*pagP* strain elicited one of the strongest responses of the mutants we tested, presumably because its deletion resulted in a greater fraction of hexa-acylated lipid A. Under normal function, PagP is an acyltransferase that is expressed under the PhoP/Q system in response to cell damage. Mutants lacking *pagP* would not be capable of adding an acyl chain via this mechanism, thus maintaining a greater fraction of their highly immunogenic wild-type lipid A. Another interesting finding from our assay was that the strain with the *csgA* deletion, which encodes a known TLR2 agonist (24), was one of the three strains that consistently elicited the strongest dynamic response. The effect of TLR2 stimulation on dynamic behavior is rarely studied. Based on the knowledge that TLR4 stimulation increases dynamic behavior, one might expect that if an effect were to be observed, it would be that lack of stimulation of TLR2 would reduce the amount of cell movement relative to the wild type. Our results may point toward a previously unknown role for TLR2 in pathogen response of tempering neutrophil activation when detected in concert with LPS or other immunogenic molecules.

Several of the mutant strains we tested (Δ*rfbP*, Δ*rfbK*, Δ*rfc*, Δ*manA*, Δ*rfaL*, Δ*rfaG*, and Δ*rfaH* strains) lacked or possessed a truncated O antigen but produced a widely varying range of dynamic responses (data marked green and light blue in Fig. 2). We consistently observed one of the strongest responses to the Δ*rfbP* mutants. Our ranked order of chemokinesis response is in agreement the TLR4 stimulation results from antibody titer assays by Kong et al. (29) showing that an Δ*rfbP* strain was significantly more immunogenic than Δ*rfaL*, Δ*rfaG*, and Δ*rfaH* strains. Interestingly, these authors also found that the Δ*rfc* strain caused a response similar to that elicited by the Δ*rfbP* strain, which did not translate in our migration-based assay, though the difference between the *D*_MSF_ responses to the two was smaller than for speed and ASD. Similar and relatively strong responses were observed in experiments with Δ*rfbK* and Δ*rfaL* strains, though the response to the Δ*rfaL* strain was marginally lower. While Δ*rfbP*, Δ*rfbK*, Δ*rfc*, Δ*rfaL*, and Δ*manA* strains putatively have nearly identical LPS structures (note that the Δ*rfc* strain generates a single O-antigen subunit), the Δ*rfaH* and Δ*rfaG* strains each have defects in the LPS core as well. These two strains elicited responses among the weakest found in our experiments relative to all the other strains tested. Collectively, these results indicate that elimination of the O antigen has the capacity to enhance the dynamic response (Δ*rfbP* strain), insignificantly affect it (Δ*rfbK*, Δ*rfc*, and Δ*rfaL* strains), or reduce it (Δ*manA*, Δ*rfaH*, and Δ*rfaG* strains). We posit that differences in compensatory gene expression (e.g., damage response genes) between the five O-antigen mutants expressing a normal core are responsible for the differences in dHL-60 activation. Expression of surface appendages such as curli fimbriae (composed in part of the TLR2 ligand CsgA) and flagella (made of flagellin, a TLR5 ligand) may have been affected differently between the OM mutant strains, further altering the overall response of the dHL-60s.

Finally, several of the strains used in our study constitutively produced defective lipid A (data in red in Fig. 2), which resulted in widely varying dynamic responses. The Δ*msbB* mutants lack a myristate and produce a mixture of penta-acylated and hexa-acylated lipid A as opposed to the mixture of hexa-acylated and hepta-acylated produced by their wild-type counterparts (30). Penta-acylated lipid A is less stimulatory of TLR4 than hepta-acylated lipid A (4). This mutation has been shown to significantly attenuate the bacteria and has been identified as one of the key attenuations in the tumor-targeting strain VNP20009 (21), which we tested as well. Not surprisingly, Δ*msbB* mutants elicited the weakest dynamic response according to our three metrics, each of which was significantly lower than that for at least 11 other mutant strains. Moreover, the average peak values for instantaneous speed, *D*_MSF_, and ASD were lower in response to the Δ*msbB* strain than the wild type, with statistically significant differences for the latter two. Interestingly, however, we found that the response to the Δ*msbB* strain was also significantly lower than the response to VNP20009 (similar to that to the wild type) but similar to the response to VNP20009*msbB*^+^, which demonstrates that complex alterations of gene expression are at play in this tumor-targeting strain. Indeed, as discussed below, we also found that the Δ*msbB* strain, which reportedly continues to produce the O antigen (30), was extremely susceptible to complement-mediated killing, while VNP20009 and VNP20009*msbB*^+^ were minimally affected.

Additionally, we tested a Δ*lpxO* strain, which lacks a hydroxyl group on the myristate added by MsbB, and the metabolic mutant Δ*galE* (which leads to a defect in the inner core) (Fig. 1A) and Δ*aroA* mutants, and a Δ*manC* strain. We found that the responses to these strains were each similar to the response to the wild type. The Δ*aroA* strain was chosen because it has been used as an attenuated tumor-targeting strain with reported indirect effects on LPS biosynthesis (20). We found this strain to be resistant to complement; thus, its attenuation may primarily manifest as reduced fitness and limitation to nutrient-rich environments. The Δ*manC* mutant reportedly produces normal LPS but contains less per bacterium than wild-type (31). The similarity in the responses to these two strains, however, suggests that the reduction in LPS content was relatively small, possibly reflecting neutrophils’ lack of sensitivity to changes at high concentrations (2).

### Single-cell chemotaxis to OM mutants.

We next inquired how the costimulation of TLRs and chemotaxis receptors, which inadvertently occurs in the presence of live pathogens, affects chemotaxis. To this end, we quantitated the effect of OM mutations on spatiotemporal dynamics of the dHL-60 chemotaxis response over 250 min. These long-term response analyses were facilitated by the maintenance of physical separation between bacteria and neutrophils in our microfluidic devices and continuous flow of buffer and bacterial suspension in the outer channels. The former prevents bacterial phagocytosis (in contrast to earlier microfluidic studies [14–17]), whereas the latter enables investigation of the response under quasi-steady-state conditions (in contrast to Transwell assays, wherein the waste is not removed and the metabolizable effectors are depleted). In our experiments, chemotaxis was mediated through formyl peptide detection by FPR1 and FPR2 and by gradients of C5a, which are generated by the reaction of complement component C5 with the bacterial OM (32). It is understood that TLRs do not detect gradients in effector concentration but can initiate a signaling cascade leading to neutrophil activation and enhanced chemokinesis, thus potentially affecting chemotaxis.

**FIG 3:**
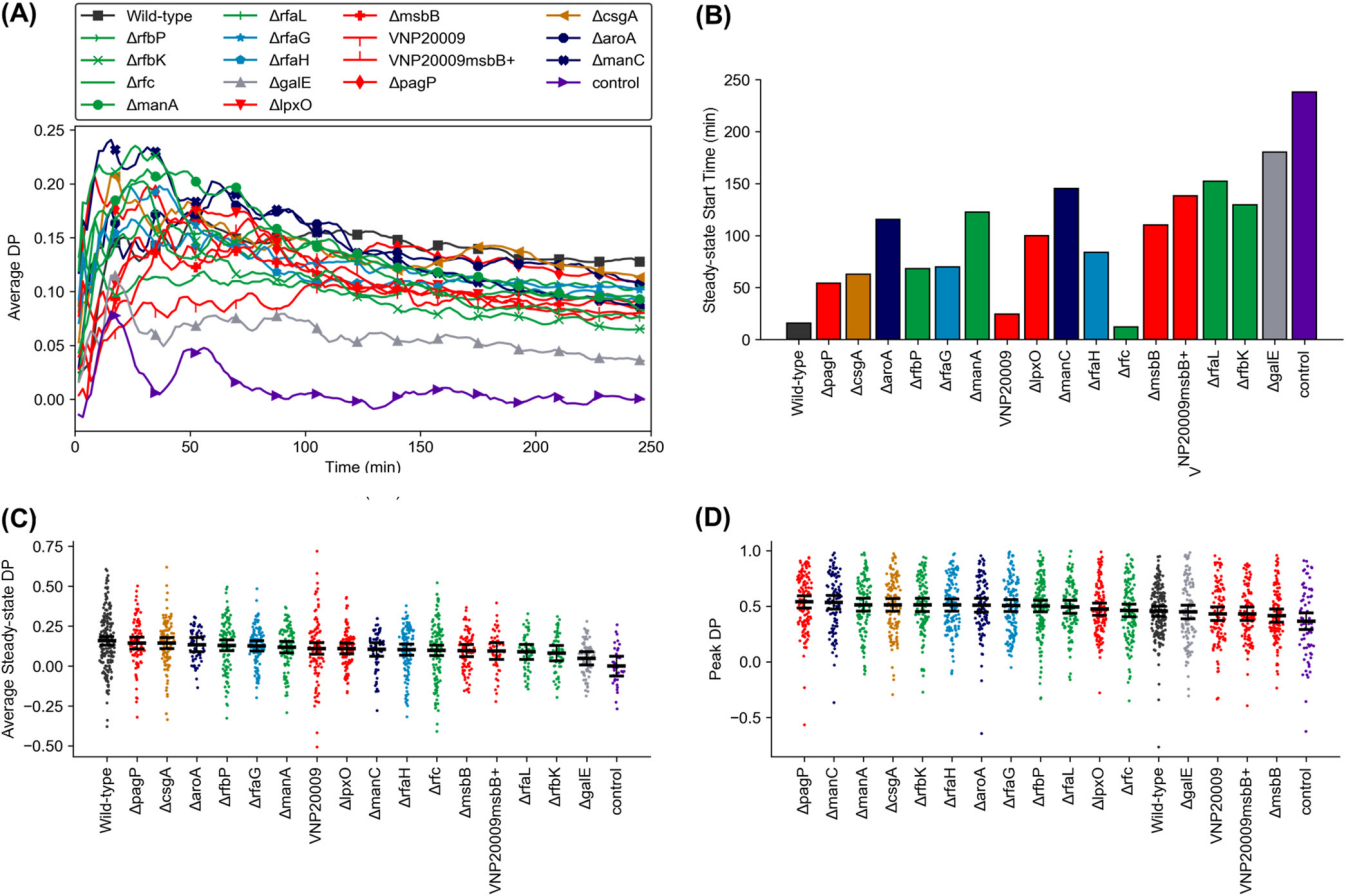
Single-cell chemotaxis response. (A) Average directional persistence (DP) of tracked dHL-60s versus time in response to stimulation with each mutant strain; (B) time from the start of the experiment to when DP becomes quasi-steady; (C) time-averaged DP over the steady-state period; (D) peak DP value excluding the first 5 min of tracking for each tracked cell. Peak values in panel D were identified after smoothing the data with a moving average of every 3 data points to reduce noise. Results in panels C and D are sorted by value. The black bars in panels C and D indicate means and 95% confidence intervals (i.e., nonoverlapping bars between any two cases indicate a significant difference with a *P* value of <0.05). All results are from collections of at least 150 total cells tracked across three or more independent experiments (except the control case with 100 cells tracked across two experiments). Marker colors correspond to the specific component of OM that was modified, as shown in Fig. 1A.

We analyzed transient chemotaxis at single-cell resolution by calculating the directional persistence (DP) of each tracked cell at each point in time:

(5) $$\text{DP}\left(t_{N}\right)=\text{direction}\times \frac{\text{displacement}}{\text{distance}}=\frac{x_{N}-x_{0}}{\left|x_{N}-x_{0}\right|}\frac{\sqrt{{\left(x_{N}-x_{0}\right)}^{2}+{\left(y_{N}-y_{0}\right)}^{2}}}{\sum_{i=1}^{N}\sqrt{{\left(x_{i}-x_{i-1}\right)}^{2}+{\left(y_{i}-y_{i-1}\right)}^{2}}}$$

where displacement is a vector between the cell’s locations at *t*_0_ and *t_N_*, and distance is the cumulative path traversed between time *t*_0_ and *t_N_*. We calculated DP for all tracked cells with respect to each bacterial strain (Fig. 3A). Due to the mathematical definition of DP, for any individual cell, the DP value at the second time point (*t*_1_) was necessarily either 1 or −1 and could then fluctuate largely in this range before converging to a steady-state mean (Fig. S4A). When averaged over all tracked cells responding to a particular strain, DP typically exhibited a transient period containing a peak response sometime after exposure to bacteria (*t *= 0), followed by a quasi-steady-state period during which DP values fell at a shallow rate and ultimately reached a steady value. We observed that DP values were somewhat dependent on the cell starting *x* location, wherein cells within 210 μm of the bacterial channel exhibit little to no chemotaxis (average DP ≈ 0.05) (Fig. S4B to E). This is expected due to the higher concentration of bacterial effectors near the bacterial channel, which presumably saturates the neutrophils’ chemoreceptors (33). Thus, we excluded cells with a starting location within 210 μm (30% of the channel width) from the wall adjacent to the bacterial channel from our analysis. We defined the onset of the quasi-steady-state period as the point in time when the DP averaged through the end of the experiment (250 min) did not deviate by more than 20% (Fig. 3B). The start of this period occurred in less than 200 min for all cases except the response to control. We then plotted the DP averaged over the steady-state period for each cell in order to determine overall strain averages (Fig. 3C). This analysis revealed that dHL-60s were more chemotactic at the single-cell level in response to wild-type bacteria than to any mutant strain, but the differences were statistically significant only with respect to the Δ*rfbK* and Δ*galE* cases, as well as for the control case. The responses to several of the mutant strains were also significantly greater than the response to the Δ*galE* strain (and control). However, the magnitude of the DP was relatively low in all cases and not significantly greater than the control for the Δ*manC* strain and for any of the strains plotted from the Δ*msbB* strain toward the right-hand side in Fig. 3C. These results indicate that the chemotaxis and chemokinesis of dHL-60s are separate, uncorrelated behaviors at the single-cell level. For instance, the Δ*rfbK* and Δ*galE* strains each caused significantly greater peak speed responses than the wild type, despite promoting low steady-state DP responses. Although those stimulating the fastest responses, the Δ*pagP*, Δ*rfbP*, and Δ*csgA* strains, also caused among the strongest responses in DP, no significant differences with respect to the wild type were observed. We wondered whether the lack of differences in DP might be due to its analysis being performed at steady state after the cells had adapted to the higher concentration of effectors in the experiment. We therefore also plotted the peak DP of each cell after the first 5 min of data collection to neglect the necessarily large initial |DP| (Fig. 3D). While the peak DP values were much larger in magnitude and the ranked order of averages differed from those of the steady-state analysis, the only significant difference existed between the Δ*pagP* and Δ*msbB* strains. The peak DP in our positive-control experiments was higher in response to fMLP, alone or in combination with C5a, than to any bacterial strain (Fig. S3C and D). These data suggest that chemotaxis response is relatively insensitive to changes in the OM. Thus, chemotaxis and chemokinesis are uncorrelated and should be both evaluated for comprehensive single-cell response analysis.

10.1128/mSphere.01012-20.4 FIG S4 Chemotaxis response in space and time. (A) Directional persistence (DP) of all cells tracked in a representative wild-type experiment, with the thick gray line showing the experiment average. (B and C) The DP of each tracked cell from all experiments as a function of the cell’s normalized starting location (x*, measured relative to the left-hand channel wall, which is adjacent to the bacteria) for all dHL-60 (B) and primary human neutrophil (C) experiments. (D and E) The DP of all cells was averaged from x* to the right-hand channel wall (black) or from the left-hand channel wall to x* (red), and the moving average from x* through x* + 0.1 for all (blue) for all dHL-60s (D) and primary human neutrophils (E). Data show that cells with a starting location of x* ≤ 0.3 (*x* ≤ 210 μm) have limited DP; thus, they were excluded from the DP analysis presented in this work. Download FIG S4, EPS file, 0.6 MB. Copyright © 2021 Leaman et al. 2021 Leaman et al. https://creativecommons.org/licenses/by/4.0/ This content is distributed under the terms of the Creative Commons Attribution 4.0 International license.

### Primary human neutrophil dynamic responses are consistent with the dHL-60 responses.

Differentiated HL-60 cells have been widely adopted as a surrogate for neutrophil behavior, and TLR2 and TLR4 ligands have been shown to have similar effects on cytokine expression in dHL-60s and primary human neutrophils (25, 34). Given the complexity of the response to live pathogens, we asked if our results in chemokinetic behavior with dHL-60 cells would be consistent with primary human neutrophil responses. We performed experiments with a subset of mutant strains to evaluate the response of primary human neutrophils. In addition to the parental wild-type strain, we chose Δ*pagP*, Δ*rfbP*, and Δ*csgA* strains in order to determine if the significantly higher dynamic responses to these mutants (Fig. 2) are also observed in primary neutrophils. We also chose the tumor-targeting strain VNP20009 due to its translational relevance and its derivative VNP20009*msbB*^+^, along with a Δ*msbB* strain, to elucidate the effects of MsbB-mediated lipid A acylation specifically. Finally, we selected Δ*manC* and Δ*galE* strains as potential attenuated-strain candidates for biomedical applications. In the latter case, Δ*galE* strains have been used as live vaccines (35). We initially envisioned the Δ*manC* strain, which reportedly synthesizes less LPS than wild-type strains but still produces the full-length O antigen (31), as possibly being a strong parental strain candidate for the development of new therapeutic or vaccine strains, though our results later suggested that it is not significantly attenuated (Fig. 2).

**FIG 4:**
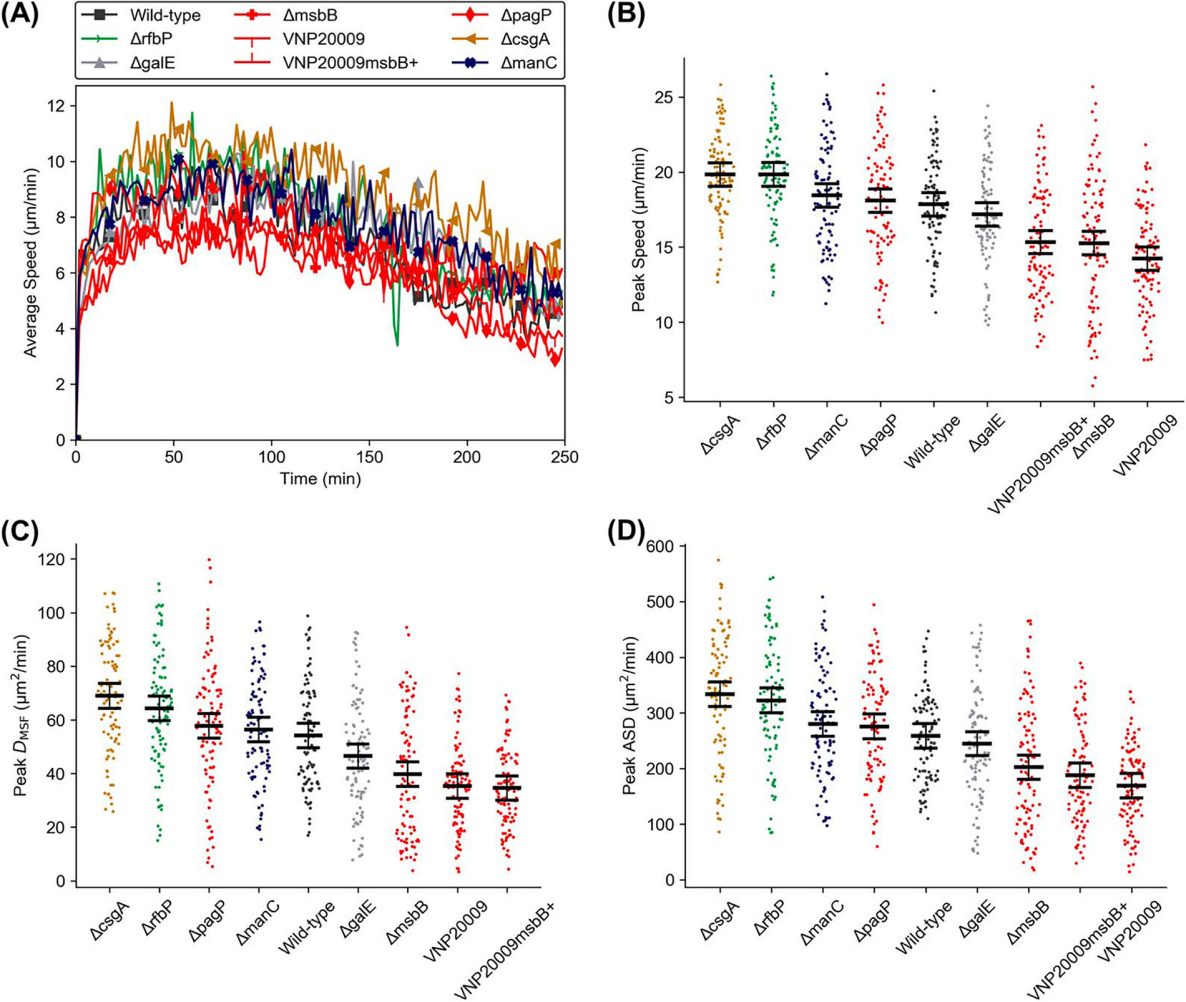
Migratory response of primary human neutrophils to mutants. (A) Speed versus time of primary neutrophils responding to a subset of *S.* Typhimurium mutant strains; (B to D) scatterplots (sorted by mean values) showing peak speed, peak *D*_MSF_, and peak ASD, respectively. The black bars in panels B, C, and D indicate means and 95% confidence intervals (i.e., nonoverlapping bars between any two cases indicate a significant difference with a *P* value of <0.05). All results are from two independent experiments with at least 50 cells tracked per experiment. Marker colors correspond to the specific component of OM that was modified, as shown in Fig. 1A.

In general, neutrophil dynamics in response to stimulation with the tested bacterial strains were found to be similar to that of dHL-60s. Speed rapidly increased with time upon the introduction of bacteria into the device, peaking within about 100 min (Fig. 4A). Notably, Δ*csgA* and Δ*rfbP* strains both elicited relatively strong responses in speed (Fig. 4B), *D*_MSF_ (Fig. 4C), and ASD (Fig. 4D). The Δ*manC* strain also provoked a similar speed response but exhibited *D*_MSF_ and ASD values that were significantly lower than those for the Δ*csgA* strain, as was the case with dHL-60s. The Δ*pagP* mutant also did not stimulate cell migration as strongly relative to other mutants as it did in dHL-60 experiments. Consistent with the results from the dHL-60 experiments, the dynamic responses to the Δ*msbB* strain and VNP20009*msbB*^+^ were among the lowest according to all three analysis metrics. The response to VNP20009 was similarly very low, although the strain prompted a response similar to that of the wild type in dHL-60 experiments. DP did not differ statistically significantly between the responses to any strain for primary human neutrophils (Fig. S5).

10.1128/mSphere.01012-20.5 FIG S5 Directional persistence of primary human neutrophils. (A) Steady-state DP, with each data point representing the DP for an individual cell averaged over the steady-state period, and (B) peak DP, identified after smoothing data by a moving average of every 3 data points and excluding data from <5 min. Error bars show 95% confidence intervals. Download FIG S5, EPS file, 0.2 MB. Copyright © 2021 Leaman et al. 2021 Leaman et al. https://creativecommons.org/licenses/by/4.0/ This content is distributed under the terms of the Creative Commons Attribution 4.0 International license.

### Altered OM structure significantly alters bacterial survival in the presence of neutrophils.

**FIG 5:**
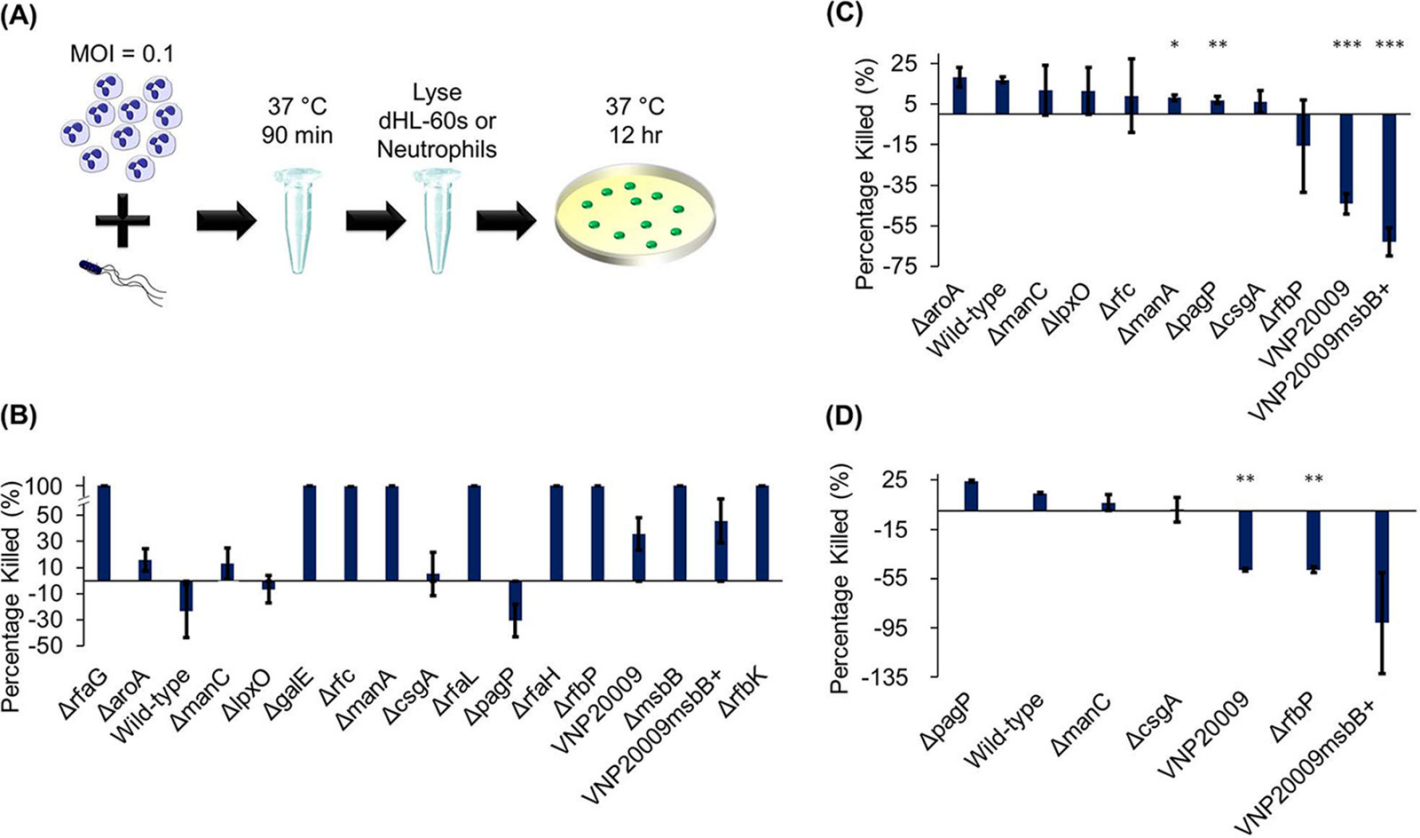
Bacterial survivability during coincubation with neutrophils and in human serum. (A) Experimental design of the neutrophil killing assay; (B) percent reduction of viable bacteria in control samples incubated in human serum relative to the starting number (viable number preincubation); (C) percent reduction in numbers of viable bacteria after incubation with dHL-60s relative to control samples (no dHL-60); (D) percentage reduction in numbers of viable bacteria after incubation with primary human neutrophils relative to control samples. *, *P* < 0.05; **, *P* < 0.01; ***, *P* < 0.001 relative to the wild type. Data were collected from at least 3 independent experiments with dHL-60s or from 2 independent experiments with primary human neutrophils.

We complemented our chemokinesis and chemotaxis findings with bacterial killing assays in human serum in order to assess the effect of OM mutation on bacterial survivability and gain a more comprehensive insight into the overall host-pathogen interactions to inform the future rational design of strains for therapeutic applications. In parallel with each microfluidic assay, we coincubated dHL-60s or neutrophils with bacteria at a multiplicity of infection (MOI [bacterium-to-cell ratio]) of 0.1 for 90 min and determined the fraction of bacteria killed through serial dilution and plating (Fig. 5A; also, see Materials and Methods). The percentage of bacteria killed was calculated from the difference between the number of CFU in controls (CFU_control_, no neutrophils) and the number of CFU from coincubated bacterium-neutrophil samples (CFU_neutrophil_) relative to the controls:

(6) $$\text{Percent}\;\text{killed}=\frac{\left({\text{CFU}}_{\text{control}}-{\text{CFU}}_{\text{neutrophil}}\right)}{{\text{CFU}}_{\text{control}}}\text{×100}$$

Prior to the neutrophil killing assay, we first examined survivability in human serum by calculating the difference between pre- and postincubation CFU counts in the experimental medium alone, which contained 10% human serum. Virtually all (>99.9%) bacteria of the Δ*rfaG*, Δ*galE*, Δ*rfaL*, Δ*rfaH*, Δ*msbB*, and Δ*rfbK* strains were killed by the experiment medium (Fig. 5B), obviating comparison between incubation with and without dHL-60s (Fig. 5C). For Δ*rfc*, Δ*manA*, and Δ*rfbP* strains, a small fraction of bacteria (up to about 1%) survived incubation in the experimental medium. The majority of the 7 remaining mutant strains and wild-type bacteria survived. The dHL-60 killing assay was performed for these 11 strains. On average, coincubated samples contained 17% fewer viable wild-type bacteria than control samples that contained bacteria alone (Fig. 5C). Similar numbers were found for the Δ*aroA* strain, while only around 10% fewer viable bacteria were measured for the Δ*manC* and Δ*lpxO* strains. The Δ*csgA* and Δ*pagP* strains were mostly unaffected by the presence of neutrophils.

Interestingly, VNP20009 and VNP20009*msbB*^+^ were found in significantly greater numbers (44% and 63%, respectively) when incubated with cells than in control cases. One of the roles of LPS is the protection of bacteria against antimicrobial complement proteins present in serum (3, 29). The Δ*msbB* strain showed the lowest survival rate of all mutants tested. Interestingly, VNP20009 survived relatively well (64%) in the medium, despite lacking a functional *msbB* gene. Likewise, VNP20009*msbB*^+^ survived at a similar rate (54%), despite its *msbB* gene being restored. Our findings suggest that *S.* Typhimurium can compensate for the lack of the *msbB* gene and adapt to survive in the host. VNP20009 was isolated as a hyperinvasive strain after random mutagenesis (21). These results are indicative of the complex nature of VNP20009 alteration and are in agreement with our findings from the chemokinesis assays, which suggested that these strains were more stimulatory than the Δ*msbB* mutant but less stimulatory than many other mutant strains. The biological mechanism responsible for the observed behavior will be explored in the future. It is also noteworthy that the Δ*manC* strain survived incubation in serum at a rate comparable to that of the wild type (91% and 93%, respectively), which is consistent with our findings with respect to chemokinesis. Results using primary human neutrophils were in good agreement with those obtained with dHL-60s (Fig. 5D).

## DISCUSSION

In this study, we quantitatively analyzed the chemokinesis and chemotaxis behavior of dHL-60s and primary human neutrophils at single-cell resolution in response to a library of *S.* Typhimurium OM mutants. Taken together, our three dynamic metrics—speed, *D*_MSF_, and ASD—provided comprehensive quantitative insight into the activation and chemokinesis elicited by each strain (Fig. 2 and 4B). Cell speed is a means by which to analyze the level of cell dynamicity at any point in time. The peak speed value did not depend on the cell distance from the channel wall (Fig. S6), demonstrating that the spatial gradient of the bacterial effectors did not affect the cell speed. Temporal plotting of the cell speed (Fig. 2A and 4A) clearly demonstrated a sharp increase in cell movement upon stimulation with bacteria and proved to be an effective tool to discriminate between responses to various mutant strains. Over time, cell speed decreased, likely indicating cell adaptation to chemical effectors produced by the bacteria (36). In contrast, *D*_MSF_ provides a cumulative measure of the cell chemokinesis. Given prior work showing that chemokinetic behavior is enhanced by stimulation with LPS, we expected that such a measure could be used to distinguish differences in cell activation (2). Indeed, the reduced noise in this cumulative measure allows clearer distinction of the chemokinetic responses to various mutants, compared to the instantaneous speed (Fig. 2C and D and 4C). The ASD complements both metrics as an intermediary between cell speed and *D*_MSF_. In contrast to the instantaneous speed, which represents a single time interval, and *D*_MSF_, which represents cumulative duration, ASD clearly indicates the cell dynamic behavior over a binned period (Fig. 2F and 4D). Using the square of the cell step size (equation 4) helps to reveal differences in responses, and binning of the data filters noise, producing curves that are smooth (relative to the speed curves) with more clearly separated peaks (relative to the *D*_MSF_ curves), as shown in Fig. 2E. Together, these three metrics facilitated a clear comparison of the dynamics of the chemokinesis of dHL-60s and primary neutrophils in response to each strain, elucidating those that are distinct and responses that are similar in strength.

10.1128/mSphere.01012-20.6 FIG S6 Neutrophil peak migration speed did not depend on the cell distance from the bacterial channel wall. The peak speed of each tracked cell as a function of normalized *x* location (x*) in all (A) dHL-60 experiments, and (B) primary human neutrophil experiments. Download FIG S6, EPS file, 0.4 MB. Copyright © 2021 Leaman et al. 2021 Leaman et al. https://creativecommons.org/licenses/by/4.0/ This content is distributed under the terms of the Creative Commons Attribution 4.0 International license.

Quantitation of the chemotaxis response at single-cell resolution reveals a lack of correlation between ranked DP values (Fig. 3) and rankings of the dynamic responses of the dHL-60 cells, which were highly gradated. The lack of gradation in chemotaxis response to various strains is in agreement with previous findings that neutrophils perform robust and uniform chemotaxis over a wide range of PAMP gradients (36, 37). While this result was not necessarily surprising, it also was not requisite. In all cases, directional migration must be mediated by GPCRs responding to bacterial proteins and complement components that are not directly affected by the mutations we employed. It would be reasonable to expect that stronger dynamic responses, such as those to the Δ*pagP* or Δ*csgA* strain, could also influence chemotaxis metrics such as DP. Stronger dynamic behavior could reasonably lead to a lower DP because a greater overall distance is traveled, or it could augment overall neutrophil accumulation at the site of bacterial colonization (i.e., the channel wall closest to bacteria in our case). Our microfluidic assay provided a unique opportunity for simultaneous investigation and direct comparison of activation (the chemokinesis) and chemotaxis to live pathogens within the same experiment.

**FIG 6:**
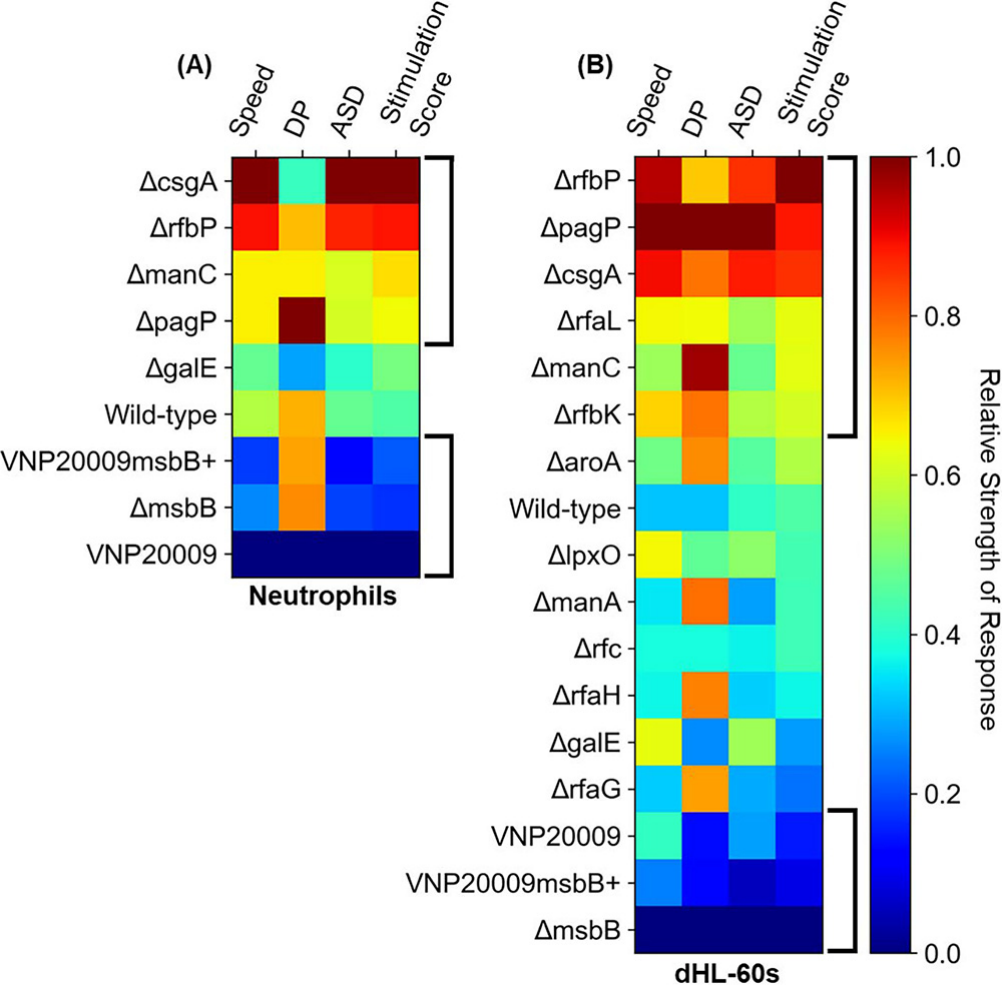
Ranking the migratory responses of dHL-60s and primary human neutrophils. Heat maps showing population-averaged peak speed, peak directional persistence (DP), peak average squared displacement (ASD), and peak stimulation score for primary human neutrophils (A) and dHL-60s (B), each scaled and normalized to range from 0 to 1 based on their respective minimum and maximum values. Stimulation score is calculated as max[DP(*t*)×ASD(*t*)]. The peak values were identified after data were smoothed by a moving average of every 3 data points. The plotted peak DP values were identified after excluding the first 5 min of tracking data (average values shown in Fig. 3D and in Fig. S5B). The scores included in the bracketed regions were significantly different from one another (*P* < 0.05).

In order to integrate our analyses of dynamics of chemokinesis and chemotaxis and rank the overall strength of the responses to various mutant strains, we calculated a stimulation score, defined as the product of ASD and DP at each point in time for each cell. We chose the ASD because it accurately represents the dynamics of chemokinesis (i.e., less susceptible to noise than speed but more representative of temporal changes than *D*_MSF_). Multiplication with DP both scales the ASD according to the level of directedness of migration and adds a sign (i.e., cells moving away from the bacteria have a negative stimulation score). A single stimulation score value was determined for each cell by taking the peak of a moving average of every three successive scores in time. For reference, averages and standard deviations for cell dynamics metrics, DP, and stimulation scores in response to each strain are included in Table S2. Heat maps showing the average responses of both primary neutrophils and dHL-60s sorted by stimulation score (Fig. 6A and B) demonstrate good agreement between the two data sets. Notably, there exist three matching “zones” between the two, with Δ*csgA*, Δ*rfbP*, Δ*manC*, and Δ*pagP* strains populating the first, with the highest stimulation scores, wild-type and Δ*galE* strains making up the second, and the three *msbB*-related mutants, VNP20009, VNP20009*msbB*^+^, and the Δ*msbB* strain, making up the third, with the lowest scores. In both cases, each score in the first zone is significantly higher than each score in the third zone.

10.1128/mSphere.01012-20.9 TABLE S2 Average and standard deviation of metrics quantifying responses to each strain. Note that the DP values and stimulation scores were calculated excluding cells with a starting location within 210 μm of the wall adjacent to the bacterial channel. Peak DP was identified excluding the first 5 min of tracking. Stimulation scores for dHL-60s and neutrophils were each normalized to their respective maximum stimulation score and scaled to a range between 0 and 1. Download Table S2, PDF file, 0.09 MB. Copyright © 2021 Leaman et al. 2021 Leaman et al. https://creativecommons.org/licenses/by/4.0/ This content is distributed under the terms of the Creative Commons Attribution 4.0 International license.

10.1128/mSphere.01012-20.7 FIG S7 Actual and estimated marginal means with cell density as a covariate. The actual means and estimated marginal means from an ANCOVA with cell density as a covariate for ASD for dHL-60s (A) and primary neutrophils (B) and for peak DP for dHL-60s (C) and primary neutrophils (D), excluding the first 5 min of data. Error bars show 95% confidence intervals. Download FIG S7, EPS file, 0.1 MB. Copyright © 2021 Leaman et al. 2021 Leaman et al. https://creativecommons.org/licenses/by/4.0/ This content is distributed under the terms of the Creative Commons Attribution 4.0 International license.

Compared to the commonly used activation metric of peak speed, the stimulation score provides a more comprehensive ranking mechanism, as it combines a more gradated measure of chemokinesis (i.e., ASD) with a measure of chemotaxis response (i.e., DP). We posit that the newly defined stimulation score could be used for initial *in vitro* screening of neutrophil response to new therapeutic or vaccine strains. As an example, our stimulation scores with respect to wild-type, Δ*aroA*, and Δ*rfaG* strains agree with *in vivo* cytokine responses reported by Frahm et al. (38). The complexities in immune response illuminated by our assay are also interesting. For instance, we found that the Δ*rfc* strain, shown to be avirulent in mice, stimulated levels of chemokinesis and a stimulation score (0.43) similar to those of the highly virulent wild-type strain (0.45) (39).

Quantitating single-cell responses demonstrated that the dynamics of neutrophil chemokinesis in response to bacterial stimulation is highly gradated depending on specific mutations, being significantly augmented or diminished relative to that of the wild type. Importantly, we found that the deletion of genes with putatively similar effects could manifest as significantly different dHL-60 responses, such as in the cases of Δ*rfbP* and Δ*manA* strains, each of which lacks the O antigen but elicited some of the strongest and weakest dynamic responses, respectively. Likewise, Δ*pagP* and Δ*msbB* strains, in which the deleted genes normally acylate lipid A, had divergent effects. In contrast, steady-state chemotaxis at the single-cell level was almost unaltered by the mutations. In comparison to positive-control experiments with fMLP and C5a, bacterial mutants can induce higher or lower chemokinetic responses with greater gradation than even order-of-magnitude changes in these effector concentrations, while chemotaxis was more robust in the controls, demonstrating a striking role for structural mutations in affecting chemokinesis (Fig. S3). These findings reaffirm the importance of using whole bacteria when evaluating the neutrophil response. In summary, we present a high-spatiotemporal-resolution microfluidic assay to comprehensively study transient and steady-state neutrophil migratory responses to live bacteria. We report novel findings with respect to the effects of the OM structure of Gram-negative pathogens on neutrophil recruitment (i.e., chemotaxis) and activation (i.e., chemokinesis) and *in vivo* survivability, providing additional insight both for basic research in infectious disease and for the rational design of therapeutic strains. Furthermore, the microfluidic assay combined with the neutrophil activation metrics reported herein will serve as a novel tool to interrogate the cell decision-making process in a variety of other host-pathogen interactions.

## MATERIALS AND METHODS

### Microfluidic device fabrication.

Microfluidic devices were fabricated based on our previously developed methods (26). Briefly, poly(dimethyl) siloxane (PDMS) (Dow Corning, Auburn, MI) mixed at a 10:1 ratio (base to curing agent) was used to spin-coat 75- by 50-mm glass slides at 450 rpm for 30 s and cured at 70°C for 4 h to form an approximately 300-μm-thick film. Three rectangular regions were cut out and peeled off each glass slide, followed by cleaning with soap, acetone, isopropanol, and deionized (DI) water. The slides were then air plasma treated (200 millitorrs, 18 W) for 40 s, immediately followed by a 10-min surface functionalization treatment using a 1% solution of 3-(trichlorosilyl) propyl methacrylate (TPM; MilliporeSigma, St. Louis, MO) in mineral oil. The TPM-treated slides were then washed with isopropanol, and the slides were baked at 95°C for 30 min. A solution of polyethylene glycol diacrylate (PEG-DA; molecular weight [MW], 700 Da; MilliporeSigma) in phosphate-buffered saline (PBS) was supplemented with 2-hydroxy-4′-(2-hydroxyethoxy)-2-methylpropiophenone (MilliporeSigma) dissolved in 70% ethanol to a final concentration of 0.05% (wt/vol). This solution was photopolymerized on the glass surface with a UV dose of 486 mJ/cm^2^ through a custom photomask to create microchannels (700-μm width and spacing) in each rectangular region. The PEG-DA-coated slides were soaked in 70% ethanol for at least 36 h prior to use, replacing the ethanol solution at least once during that time. Prior to assembly, the devices were washed in DI water and assembled using a PDMS layer and Plexiglass plates. The devices were pressure sealed, which led to a final PEG-DA channel height of about 100 μm.

### HL-60 cell culture and differentiation.

HL-60 cells (CCL-240; American Type Culture Center [ATCC], Manassas, VA) were cultured in Iscove’s modified Dulbecco’s medium (IMDM; HyClone, Logan, UT) supplemented with 10% fetal bovine serum (HyClone) at 37°C in a humidified, 7.7% CO_2_ incubator. The cell density was maintained between 1.0 × 10^5^ and 5.0 × 10^5^ cell/ml at all times. Cells were differentiated into a neutrophil-like phenotype by diluting established cultures to a density of 1.25 × 10^5^ cell/ml and supplementing the medium with dimethyl sulfoxide (DMSO; ATCC) to a final concentration of 1.56% (vol/vol). Differentiated HL-60 cells (dHL-60s) were harvested 5, 6, or 7 days after the addition of DMSO. Cells that were used in experiments had been passaged 2 to 4 times from the original ATCC stock.

### Sources of mutant strains and bacterial culture.

Unless otherwise noted, the mutant strains were obtained from the library of single-gene deletion mutants constructed by Porwollik et al. (40). These were acquired from the following, provided by BEI Resources NIAID, NIH: Salmonella enterica subsp. *enterica*, strain 14028s (serovar Typhimurium) single-gene deletion mutant library, plate 005/006_Kan, NR-29401; plate 011/012_Kan, NR-29404; plate 015/016_Kan, NR29406; plate 019, 020_Kan, NR-29408; plate SGD_029/030_Kan, NR-42825; plate SGD_043/044_Kan, NR-42832; plate SGD_152/153_Kan, NR-42847; and plate SGD_156/157_Kan, NR-42849. The parental strain, *S.* Typhimurium 14028s, the Δ*msbB* strain, and VNP20009*msbB*^+^ were gifts from the Scharf Lab (Virginia Tech), and VNP20009 (ATCC 202165) was obtained from the ATCC. Note that VNP20009*msbB*^+^ also expressed a restored *cheY* gene (23, 41), but we do not expect that this impacted dHL-60 or neutrophil behavior. For each experiment, a single colony of the desired bacterial strain was selected from a lysogeny broth (LB; 1% tryptone, 0.5% yeast extract, 1% NaCl) agar plate culture and used to inoculate 10 ml LB (supplemented with 35 μg/ml kanamycin for the library strains). The bacteria were then cultured overnight at 37°C and 100 rpm in a 125-ml smooth-bottom flask.

### Primary human neutrophil isolation.

Primary human neutrophils were collected from three healthy volunteers aged 18 years or older. All work with primary neutrophils was approved by the Institutional Review Board (IRB) office at Virginia Tech (IRB number 17-937). Neutrophils were isolated from blood samples using Polymorphprep (Alere Technologies AS, Oslo, Norway) according to the manufacturer’s instructions (including red blood cell lysis), except that Hanks’ balanced salt solution (HBSS; HyClone) without calcium or magnesium was used in place of HEPES-buffered saline. The cells were finally suspended in a solution of HBSS supplemented with 10% human AB serum (HS; Corning Inc., Corning, NY).

### Microfluidic experiments.

Microfluidic channels were coated with 5 μg/ml fibronectin (Sigma, St. Louis, MO) in Dulbecco’s PBS (DPBS) without calcium or magnesium (HyClone) for 1 h at 37°C, followed by a rinse with HBSS with 10% HS just prior to experiments. For dHL-60 experiments, cells were harvested by centrifugation at 200 × *g* for 7 min, washed once in HBSS, and resuspended at a final concentration of about 3 × 10^6^ cell/ml in HBSS with 10% HS. Primary human neutrophils were isolated as described above and likewise diluted to a final concentration of approximately 3 × 10^6^ cell/ml. Cells were then injected into the center channel of the microfluidic devices preloaded into a microscope incubator at 37°C and 5% CO_2_. After cell suspensions were flowed in, inlet and outlet tubes for cell channels were clamped to prevent flow. The overnight bacterial cultures were then harvested by centrifugation at 1,700 × *g* for 5 min, washed once in PBS, and finally resuspended in HBSS with 10% HS at a final optical density at 600 nm (OD_600_) of 2.0. The bacterial suspensions were then loaded into 1-ml disposable syringes, connected to tubes supplying one side channel of each microfluidic device, and loaded into an automated syringe pump (Harvard Apparatus, Holliston, MA) along with syringes containing sterile medium supplying the opposite side channel of each device. Once bacteria were introduced into their respective channels, time-lapse imaging of the center channels (containing dHL-60s or neutrophils) at an interval of 1.75 min for a total of 250 min commenced. Both bacteria and media were flowed through the devices at a constant rate of 15 nl/min for the duration of experiments. Positive-control experiments were performed according to the methods above, except that a 1, 10, or 100 nM solution of fMLP (MilliporeSigma, St. Louis, MO), C5a (R&D Systems, Minneapolis, MN), or a combination of the two (100 nM C5a and 1, 10, or 100 nM fMLP) in HBSS with 10% HS was flowed through one flow channel of the microfluidic device rather than bacteria.

To confirm that stochastic differences in the number of cells seeded into the channel did not affect the outcome, we performed an analysis of covariance (ANCOVA) for ASD and DP with cell density in the microfluidic device as a covariate, finding minimal changes in marginal means and confidence intervals with respect to the unadjusted results (Fig. S7).

### dHL-60/neutrophil killing and serum survival assays.

For killing assays, dHL-60 cells, primary human neutrophils, and bacteria were prepared as described above for the microfluidic assays, except that the leukocytes were suspended at a final concentration of 5 × 10^6^ cell/ml, and bacteria were diluted to a final OD_600_ of 0.001 (corresponding to approximately 5 × 10^5^ CFU/ml). HBSS with 10% HS was used as the medium for the assays. Aliquots (50 μl) of bacteria and dHL-60s or neutrophils were combined in 1.5-ml microcentrifuge tubes. Controls were prepared by substituting 50 μl of sterile medium for the dHL-60 or neutrophil suspension. The tubes were taped on their sides to the platform of a Belly Button shaker (IBI Scientific, Dubuque, IA) set to approximately 60% maximum speed and incubated at 37°C for 90 min. The dHL-60s or neutrophils were then lysed by adding 100 μl of 1% Triton-X (Fisher Scientific, Pittsburgh, PA) in PBS to each tube and incubating at room temperature for at least 10 min. Each tube was then shaken vigorously by hand, and the solutions were diluted appropriately and plated onto 1.5% LB agar plates. After about 12 h incubation at 37°C, the number of colonies was counted and assumed to correspond to the number of CFU in the plated suspension. All experiments were performed in triplicate. In parallel with the killing assays, samples of the initial bacterial suspensions were diluted and plated in duplicate in order to quantify the concentration of viable bacteria at the start of each assay and to calculate the fraction killed by complement proteins (control samples).

### Statistics and data analysis.

For the chemokinetic behavior analyses, a minimum of 50 cells from each experiment were manually tracked using ImageJ (National Institutes of Health, Bethesda, MD). Only cells with spread morphology that did not appear obviously dead, nondifferentiated (in the case of dHL-60s), fixated on debris adhered to the glass surface, or otherwise incapable of migrating were selected for tracking. Tracking data, which consisted of cell *x* and *y* coordinates at each point in time, were processed using a custom Python script. Instantaneous speed data were smoothed using a moving average of every three data points to reduce noise.

Statistical significance for all migration metrics was determined by performing multiple comparisons using Tukey’s honest significance test (α = 0.05). Statistical significance and *P* values for killing and survival assay data were determined using pairwise *t*-tests. At least three independent experiments were performed for each strain.
