# Supplementary material for: Outer Membrane Structural Defects in Salmonella enterica Serovar Typhimurium Affect Neutrophil Chemokinesis but Not Chemotaxis
Source: mSphere. 2021 Feb 24;6(1):e01012-20. doi: 10.1128/mSphere.01012-20 (PMC8544890; doi:10.1128/mSphere.01012-20)
Supplement: TABLE S1 [file msphere.01012-20-st001.pdf]

Group	Strain	Protein(s) Affected	Description	Reference
**Parental**	14028s	N/A	Wild-type parental strain; highly virulent, highly inflammatory	N/A
**Metabolic Attenuation**	Δ*aroA*	3-phosphoshikimate 1-carboxyvinyltransferase	Mutant lacks enzyme connecting glycolysis to aromatic amino acid synthesis; expression of a variety of genes is affected	(1)
**Defect in LPS Core**	Δ*galE*	UDP-galactose-4-epimerase	Mutant lacks the enzyme required to metabolize galactose, a core sugar in LPS	(2, 3)
	Δ*rfaG*	Glucosyltransferase I	Mutant has highly truncated LPS; no outer core can be added to the inner core; there is no O-antigen	(1, 4)
	Δ*rfaH*	Transcriptional antiterminator	Mutant has defective core LPS and lacks the O-antigen	(5)
**Structural Change to Lipid A**	Δ*pagP*	Palmitoyl transferase for lipid A	Adds a palmitate to lipid A in response to membrane damage; lack of gene results in more hexa-acylated LPS (more immunogenic)	(6–9)
	Δ*msbB*	(KDO)2-(lauroyl)-lipid IVA acyltransferase	Transfers myristate to lipid A; thought to be a significant factor in attenuation of tumor-targeting VNP20009	(10, 11)
	Δ*lpxO*	dioxygenase for lipid synthesis	Adds a hydroxyl group to myristate at 3'; altered structure may have implications for TLR4 activation	(9)
	VNP20009	PurI, MsbB	PurI mediates purine biosynthesis; MsbB adds terminal myristate to lipid A. Administered to humans safely in two Phase I clinical trials	(10, 12)
	VNP20009 *msbB*$^{+}$	(KDO)2-(lauroyl)-lipid IVA acyltransferase	Used to elucidate the role of other mutations in VNP20009	(13)
**Defect in O-antigen**	Δ*rfc*	O-antigen polymerase	O-antigen is not synthesized to its full length	(5)
	Δ*rfbK*	phosphomannomutase	Mutant has no O-antigen	(14)
	Δ*rfbP*	O-antigen transferase	Mutant has no O-antigen	(15)
	Δ*manA*	mannose-6-phosphate isomerase	Mutant is unable to synthesize the O-antigen, but still expresses the full core lipid A	(2)
	Δ*rfaL*	O-antigen ligase	Mutant has a complete LPS core but lacks the O-antigen	(4)
**Other Mutations**	Δ*manC*	mannose-1-phosphate guanylyltransferase	Mutant produces less LPS, has improved ability to form biofilms, and induces lower reactive oxygen species (ROS) response in macrophages	(16)
	Δ*csgA*	cryptic curlin major subunit	CsgA was shown to be a TLR2 PAMP; mutants have reduced production of fimbria	(17, 18)

References

1. Felgner S, Frahm M, Kocijancic D, Rohde M, Eckweiler D, Bielecka A, Bueno E, Cava F, Abraham W-R, Curtiss R, Häussler S, Erhardt M, Weiss S. 2016. *aroA*-Deficient *Salmonella enterica* Serovar Typhimurium Is More Than a Metabolically Attenuated Mutant. MBio 7:e01220-16.
2. Collins LV, Attridge S, Hackettt J. 1991. Mutations at *rfc* or *pmi* Attenuate *Salmonella typhimurium* Virulence for Mice. Infect Immun 59:1079–1085.
3. Germanier R, Fürer E. 1971. Immunity in Experimental Salmonellosis. II. Basis for the Avirulence and Protective Capacity of *gal* E mutants of *Salmonella typhimurium*. Infect Immun 4:663–673.
4. Zenk SF, Jantsch J, Hensel M. 2009. Role of *Salmonella enterica* lipopolysaccharide in activation of dendritic cell functions and bacterial containment. J Immunol 183:2697–707.
5. Kong Q, Yang J, Liu Q, Alamuri P, Roland KL, Curtiss III R. 2011. Effect of Deletion of Genes Involved in Lipopolysaccharide Core and O-Antigen Synthesis on Virulence and Immunogenicity of *Salmonella enterica* Serovar Typhimurium. Infect Immun 79:4227–4239.
6. Dalebroux ZD, Matamouros S, Whittington D, Bishop RE, Miller SI. 2014. PhoPQ regulates acidic glycerophospholipid content of the *Salmonella* Typhimurium outer membrane. Proc Natl Acad Sci USA 111:1963–8.
7. Broz P, Ohlson MB, Monack DM. 2012. Innate immune response to *Salmonella typhimurium*, a model enteric pathogen. Gut Microbes 3:62–70.
8. Kawasaki K, Ernst RK, Miller SI. 2004. 3-O-Deacylation of Lipid A by PagL,

a PhoP/PhoQ-regulated Deacylase of *Salmonella typhimurium*, Modulates Signaling through Toll-like Receptor 4. J Biol Chem 279:20044–20048.

9. Needham BD, Carroll SM, Giles DK, Georgiou G, Whiteley M, Trent MS. 2013. Modulating the innate immune response by combinatorial engineering of endotoxin. Proc Natl Acad Sci USA 110:1464–1469.

10. Low KB, Ittensohn M, Le T, Platt J, Sodi S, Amoss M, Ash O, Carmichael E, Chakraborty A, Fischer J, Lin SL, Luo X, Miller SI, Zheng L, King I, Pawelek JM, Bermudes D. 1999. Lipid A mutant *Salmonella* with suppressed virulence and TNFα induction retain tumor-targeting in vivo. Nat Biotechnol 17:37–41.

11. Kong Q, Six DA, Liu Q, Gu L, Roland KL, Raetz CRH, Curtiss R. 2011. Palmitoylation State Impacts Induction of Innate and Acquired Immunity by the *Salmonella enterica* Serovar Typhimurium *msbB* Mutant. Infect Immun 79:5027–5038.

12. Broadway KM, Modise T, Jensen R V, Scharf BE. 2014. Complete genome sequence of *Salmonella enterica* serovar Typhimurium VNP20009, a strain engineered for tumor targeting. J Biotechnol 192:177–178.

13. Broadway KM, Suh S, Behkam B, Scharf BE. 2017. Optimizing the restored chemotactic behavior of anticancer agent *Salmonella enterica* serovar Typhimurium VNP20009. J Biotechnol 251:76–83.

14. Turner AK, Lovell MA, Hulme SD, Zhang-barber LI, Barrow PA. 1998. Identification of *Salmonella typhimurium* Genes Required for Colonization of the Chicken Alimentary Tract and for Virulence in Newly Hatched Chicks

66:2099–2106.

15. Saldías MS, Patel K, Marolda CL, Bittner M, Contreras I, Valvano MA. 2008. Distinct functional domains of the *Salmonella enterica* WbaP transferase that is involved in the initiation reaction for synthesis of the O antigen subunit. Microbiology 154:440–453.

16. Thomsen LE, Chadfield MS, Bispham J, Wallis TS, Olsen JE, Ingmer H. 2003. Reduced amounts of LPS affect both stress tolerance and virulence of *Salmonella enterica* serovar Dublin. FEMS Microbiol Lett 228:225–231.

17. Tükel Ç, Raffatellu M, Humphries AD, Wilson RP, Andrews-polymenis HL, Gull T, Figueiredo JF, Wong MH, Michelsen KS, Akçelik M, Adams LG, Bäumler AJ. 2005. CsgA is a pathogen-associated molecular pattern of *Salmonella enterica* serotype Typhimurium that is recognized by Toll-like receptor 2. Mol Microbiol 58:289–304.

18. El Hag M, Zheng F, Yangyang S, Xiao W, Yassin A, Sujuan C, Peng D, Liu X. 2017. Contribution of the *csgA* and *bcsA* genes to *Salmonella enterica* Serovar Pullorum Biofilm Formation and Virulence. Avian Pathol 9457:1–31.
