# Supplementary material for: Outer Membrane Structural Defects in Salmonella enterica Serovar Typhimurium Affect Neutrophil Chemokinesis but Not Chemotaxis
Source: mSphere. 2021 Feb 24;6(1):e01012-20. doi: 10.1128/mSphere.01012-20 (PMC8544890; doi:10.1128/mSphere.01012-20)
Supplement: TABLE S2 [file msphere.01012-20-st002.pdf]

	Strain	Speed (μm/min)	D_{MSF} (μm^2/min)	ASD (μm^2/min)	Peak DP	Stimulation Score
**dHL-60s**	Wild-type	14.23 ± 3.71	39.26 ± 18.19	194.57 ± 86.54	0.45 ± 0.23	0.45 ± 0.45
	Δ*aroA*	15.06 ± 2.55	39.34 ± 14.11	201.42 ± 63.91	0.51 ± 0.26	0.57 ± 0.38
	control	9.21 ± 2.62	17.77 ± 10.15	77.61 ± 44.69	0.37 ± 0.32	-0.46 ± 0.21
	Δ*csgA*	17.05 ± 3.05	48.88 ± 15.97	261.53 ± 87.47	0.51 ± 0.25	0.86 ± 0.50
	Δ*galE*	15.58 ± 2.51	38.48 ± 13.91	210.58 ± 72.35	0.45 ± 0.29	0.28 ± 0.40
	Δ*lpxO*	15.70 ± 2.48	36.13 ± 12.29	211.29 ± 64.72	0.47 ± 0.23	0.43 ± 0.37
	Δ*manA*	14.13 ± 3.13	32.25 ± 15.54	169.53 ± 74.19	0.51 ± 0.25	0.43 ± 0.44
	Δ*manC*	15.20 ± 2.70	38.14 ± 16.73	195.10 ± 74.60	0.53 ± 0.25	0.63 ± 0.46
	Δ*msbB*	13.43 ± 3.09	26.97 ± 10.61	153.32 ± 59.53	0.42 ± 0.24	0.00 ± 0.30
	Δ*pagP*	17.22 ± 2.88	51.34 ± 18.20	263.64 ± 94.06	0.54 ± 0.25	0.89 ± 0.47
	Δ*rfaG*	14.58 ± 2.65	32.93 ± 12.62	182.57 ± 71.81	0.51 ± 0.25	0.24 ± 0.36
	Δ*rfaH*	14.51 ± 3.32	30.58 ± 12.26	182.59 ± 80.30	0.51 ± 0.24	0.37 ± 0.41
	Δ*rfaL*	15.79 ± 2.37	38.88 ± 12.14	210.20 ± 67.99	0.49 ± 0.23	0.63 ± 0.40
	Δ*rfbK*	15.91 ± 2.88	44.16 ± 16.45	223.90 ± 81.33	0.51 ± 0.25	0.61 ± 0.48
	Δ*rfbP*	16.89 ± 3.10	48.84 ± 18.27	250.12 ± 88.29	0.50 ± 0.27	1.00 ± 0.60
	Δ*rfc*	14.70 ± 3.30	42.88 ± 19.09	189.89 ± 78.16	0.46 ± 0.27	0.43 ± 0.48
	VNP20009	15.05 ± 2.64	37.94 ± 12.65	185.54 ± 61.90	0.43 ± 0.27	0.15 ± 0.28
	VNP20009 *msbB*$^+$	14.21 ± 2.98	29.84 ± 12.65	156.02 ± 56.71	0.43 ± 0.24	0.09 ± 0.32
**Neutrophils**	Wild-type	17.85 ± 3.11	54.22 ± 19.43	264.72 ± 82.36	0.49 ± 0.30	0.45 ± 0.36
	Δ*csgA*	19.83 ± 2.80	68.97 ± 20.59	346.53 ± 107.75	0.45 ± 0.26	1.00 ± 0.56
	Δ*galE*	17.18 ± 3.27	46.53 ± 20.47	251.89 ± 102.59	0.44 ± 0.24	0.49 ± 0.36
	Δ*manC*	18.44 ± 3.48	56.39 ± 19.62	288.58 ± 99.64	0.48 ± 0.28	0.67 ± 0.43
	Δ*msbB*	15.26 ± 4.44	39.71 ± 22.88	209.42 ± 115.47	0.50 ± 0.33	0.17 ± 0.36
	Δ*pagP*	18.10 ± 3.67	57.83 ± 24.36	279.99 ± 95.52	0.53 ± 0.28	0.64 ± 0.49
	Δ*rfbP*	19.83 ± 3.15	64.35 ± 22.36	332.34 ± 107.71	0.49 ± 0.26	0.89 ± 0.50
	VNP20009	14.23 ± 3.25	35.31 ± 16.23	174.93 ± 72.98	0.40 ± 0.21	0.00 ± 0.25
	VNP20009 *msbB*$^+$	15.33 ± 3.62	34.58 ± 14.68	193.66 ± 87.05	0.49 ± 0.23	0.21 ± 0.31
